# Data on the test-retest reproducibility of streamline counts as a measure of structural connectivity

**DOI:** 10.1016/j.dib.2018.05.145

**Published:** 2018-06-05

**Authors:** Lena V. Schumacher, Marco Reisert, Kai Nitschke, Karl Egger, Horst Urbach, Jürgen Hennig, Cornelius Weiller, Christoph P. Kaller

**Affiliations:** a Dept. of Neurology, Medical Center–University of Freiburg, Faculty of Medicine, University of Freiburg, Breisacher Strasse 64, 79106 Freiburg, Germany; b Medical Psychology and Medical Sociology, Faculty of Medicine, University of Freiburg, Rheinstrasse 12, 79104 Freiburg, Germany; c Dept. of Neuroradiology, Medical Center–University of Freiburg, Faculty of Medicine, University of Freiburg, Breisacher Strasse 64, 79106 Freiburg, Germany; d Freiburg Brain Imaging Center, University of Freiburg, Germany; e BrainLinks-BrainTools Cluster of Excellence, University of Freiburg, Germany; f Medical Physics, Dept. of Radiology, Medical Center–University of Freiburg, Faculty of Medicine, University of Freiburg, Breisacher Strasse 60a, 79106 Freiburg, Germany

## Abstract

These data provide estimations of test-retest reproducibility of streamline counts based on diffusion weighted imaging (DWI) data using a global tractography algorithm in a sample of young healthy adults. Data on descriptive statistics and factorial analyses of within-session and between-session reproducibility in terms of intra-class correlation coefficients for the absolute agreement between measurements are provided. The effect of several exemplary methodological parameters pertaining to different steps along the tractography processing pipeline on reproducibility are considered. These data are related to the research article entitled ‘Probing the reproducibility of quantitative estimates of structural connectivity derived from global tractography’ (Schumacher et al., Neuroimage, 175 (2018) 215–229).

**Specifications Table**

**Table:** 

Subject area	*Neuroscience*
More specific subject area	*Diffusion weighted imaging and tractography*
Type of data	*Figure, table*
How data was acquired	*Diffusion-weighted MR images were acquired on a SIEMENS MR tomograph from human participants*
Data format	*Analyzed*
Experimental factors	*Type of head-coil (12-channel vs. 32-channel coil), number of reconstruction repetitions (1 vs 10 repetitions)*, *streamline selection variant (defining fuzzy versus no fuzzy borders of the seed mask; selecting streamlines that end in versus that visit a seed)*
Experimental features	*Participants were scanned twice within one week and tractography was performed in two independent tracking runs for both testing sessions using both types of head-coil. Whole-brain fiber reconstruction was carried out once with 1 repetition and once with 10 repetitions. Streamlines for connections between the seeds of the AAL atlas were selected using four different streamline selection variants (endpoint_nofuzzy; endpoint_fuzzy; visiting_nofuzzy; visiting_fuzzy).*
Data source location	*Freiburg, Germany*
Data accessibility	*Data is provided with this article*
Related research article	*Associated research article:*
	*Schumacher LV, Reisert M, Nitschke K, Egger K, Urbach H, Hennig J, Weiller C, Kaller CP. Probing the reproducibility of quantitative estimates of structural connectivity derived from global tractography. Neuroimage, 175 (2018) 215–229.*

**Value of the data**

• The data provide comprehensive information on how the test-retest reproducibility of structural connectivity is influenced by methodological parameters commonly used with fiber tractography algorithms (e.g. seed-based selection of streamlines).
• The data can inform future research using the global tractography technique to quantitatively assess differences in structural connectivity (e.g. in patient studies).
• Data is based on the common AAL brain atlas, thus allowing comparisons with other reproducibility analyses using the same atlas (e.g. for different tractography approaches).

## 1 Data

The data of this article provide information on the test-retest reproducibility of quantitative estimates of structural connectivity based on whole-brain fiber tractography of diffusion-weighted MR images using the global tractography approach by Reisert and colleagues [1]. Streamline counts (i.e. the number of reconstructed ‘fibers’) of all pairwise connections between seeds of the AAL atlas were used as the quantitative measure of structural connectivity. The data presented here describe the test-retest reproducibility of these streamline counts for both within-session (comparing two independent tracking runs of the same data) and between-session (comparing data from two independent testing sessions) measurements. Data are provided on the effect of three methodological parameters on test-retest reproducibility: type of head-coil (12-channel vs. 32-channel coil), number of reconstruction repetitions (1 vs. 10 repetitive reconstructions of streamlines), and streamline selection variant (endpoint_nofuzzy; endpoint_fuzzy; visiting_nofuzzy; visiting_fuzzy). In the related research article (Schumacher et al., Probing the reproducibility of quantitative estimates of structural connectivity derived from global tractography), analyses are restricted to one streamline selection variant (endpoint_fuzzy), whereas this data set provides reproducibility statistics for all four streamline selection variants. These variants refer to selecting streamlines based on (1) whether they end in versus visit (i.e. pass through) a seed [endpoint vs. visiting] and (2) whether the image mask of the seed is used in its original binary version (i.e. each voxel has a value of 1 if it lies inside the seed image mask or 0 if it lies outside the seed image mask; streamlines are selected based on voxels with a value of 1 for a given seed) or whether ‘fuzzy’ borders of the seed image mask are defined to select streamlines by applying a Gaussian kernel to seed image voxels, so that voxels with a given minimum probability of lying within the seed image mask are used for streamline selection [nofuzzy vs. fuzzy]. For a detailed description, see Section 2, Streamline Selection. For a visualization of exemplary connections with the four streamline selection variants, see the related research article (Schumacher et al., Probing the reproducibility of quantitative estimates of structural connectivity derived from global tractography).

**Fig. 1:**
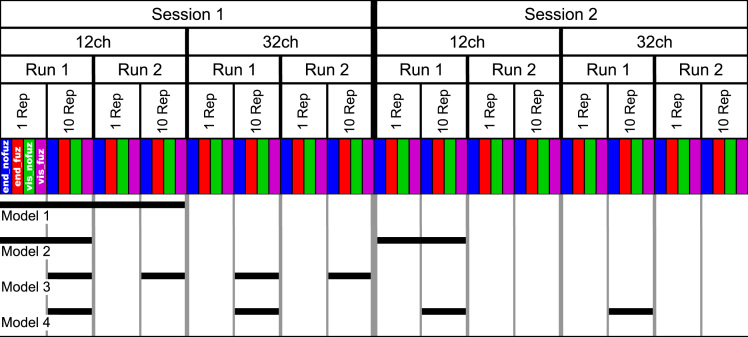
Experimental design of the data set. Diffusion-weighted imaging sequences were acquired on two separate testing sessions (*Session 1* and *Session 2*) and with a 12-channel (*12ch*) and 32-channel (*32ch*) head-coil. On each of these four resulting diffusion tensor imaging data sets, two independent runs of global tractography were performed (*Run 1* and *Run 2*). During each tracking run, streamlines were reconstructed once with 1 reconstruction repetition (*1 Rep*) and once with 10 reconstruction repetitions (*10 Rep*). For each of these tractography data sets, the selection of streamlines was performed with four different variants of selection parameters: endpoint_nofuzzy (*end_nofuz*; blue shading); endpoint_fuzzy (*end_fuz*; red shading); visiting_nofuzzy (*vis_nofuz*; green shading); and visiting_fuzzy (*vis_fuz*; purple shading), resulting in 64 subject-specific data sets of streamline counts. Statistical analyses aimed at probing the effect of number of reconstruction repetitions, type of head-coil, and streamline selection variant on within-session and between-session test-retest reproducibility, resulting in four analyses: Model 1, within-session reproducibility of *reconstruction repetitions*×*streamline selection variant*; Model 2, between-session reproducibility of *reconstruction repetitions*×*streamline selection variant*; Model 3, within-session reproducibility of *type of head-coil*×*streamline selection variant*; Model 4, between-session reproducibility of *type of head-coil*×*streamline selection variant*. Black bars indicate which sub-set of data entered these four statistical models.

The analytical design of the current data set is depicted in Fig. 1 (for further information, see Section 2, Experimental design and statistical analysis). Briefly, reproducibility statistics are provided assessing the effect of the number of reconstruction repetitions and streamline selection variants for within-session data (Model 1) and between-session data (Model 2) and assessing the effect of type of head-coil and streamline selection variants for within-session data (Model 3) and between-session data (Model 4).

**Fig. 2:**
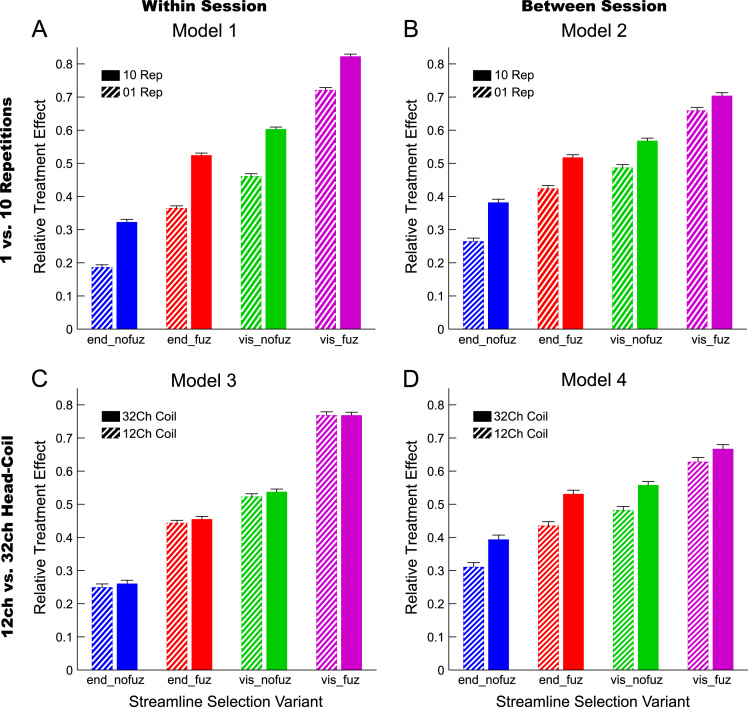
Relative treatment effects (RTEs) for the four statistical models. The RTE for a factor combination denotes the probability that a randomly chosen observation for that factor combination yields a higher ICC(2,1) value than a randomly chosen observation from the whole data set. Thus, higher RTEs indicate higher ICC(2,1) values for that factor combination, expressed as a probability value ranging from 0 to 1. Panels depict RTEs for (A) Model 1, within-session reproducibility of *reconstruction repetitions*×*streamline selection variant*; (B) Model 2, between-session reproducibility of *reconstruction repetitions*×*streamline selection variant*; (C) Model 3, within-session reproducibility of *type of head-coil* ×*streamline selection variant*; and (D) Model 4, between-session reproducibility of *type of head-coil* ×*streamline selection variant.* Error bars denote the 95% confidence interval of RTEs. End_nofuz, endpoint_nofuzzy; end_fuz, endpoint_fuzzy; vis_nofuz, visiting_nofuzzy; and vis_fuz, visiting_fuzzy streamline selection. 1 Rep, 1 reconstruction repetition; 10 Rep, 10 reconstruction repetitions. 12ch Coil, 12-channel head-coil; 32ch Coil, 32-channel head-coil.

**Fig. 3:**
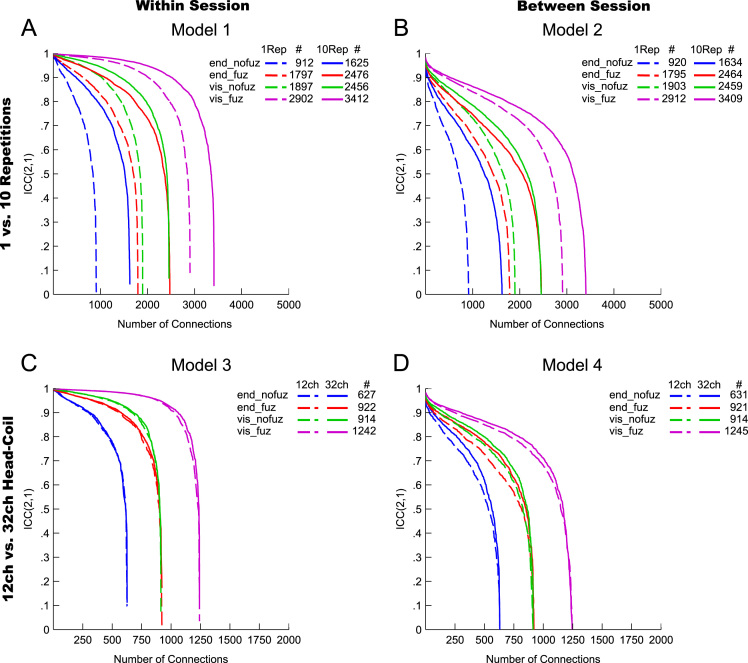
ICC(2,1) values for (A) Model 1, within-session reproducibility of *reconstruction repetitions* × *streamline selection variant*; (B) Model 2, between-session reproducibility of *reconstruction repetitions* × *streamline selection variant*; (C) Model 3, within-session reproducibility of *type of head-coil* × *streamline selection variant*; and (D) Model 4, between-session reproducibility of *type of head-coil* ×*streamline selection variant.* The *y*-axis denotes the magnitude of ICC values; the *x*-axis denotes the connections in descending order of ICC value magnitude. Dashed lines, 1 reconstruction repetition (A, B) and 12ch head-coil (C, D); solid lines, 10 reconstruction repetitions (A, B) and 32ch head-coil (C, D). Blue lines, endpoint_nofuzzy (end_nofuz); red lines, endpoint_fuzzy (end_fuz); green lines, visiting_nofuzzy (vis_nofuz); and purple lines, visiting_fuzzy (vis_fuz) streamline selection variant. #, total number of connections reconstructed in at least 95% of subjects for the given combination of streamline selection variant and reconstruction repetition (A, B) or for the given streamline selection variant across both 12ch and 32ch data (C, D). Note that the total number of connections was identical for 12ch and 32ch data because only connections found in both types of head-coil data were considered for analysis. For visualization purposes, negative ICC(2,1) values were set to zero (0.13% of values).

**Fig. 4:**
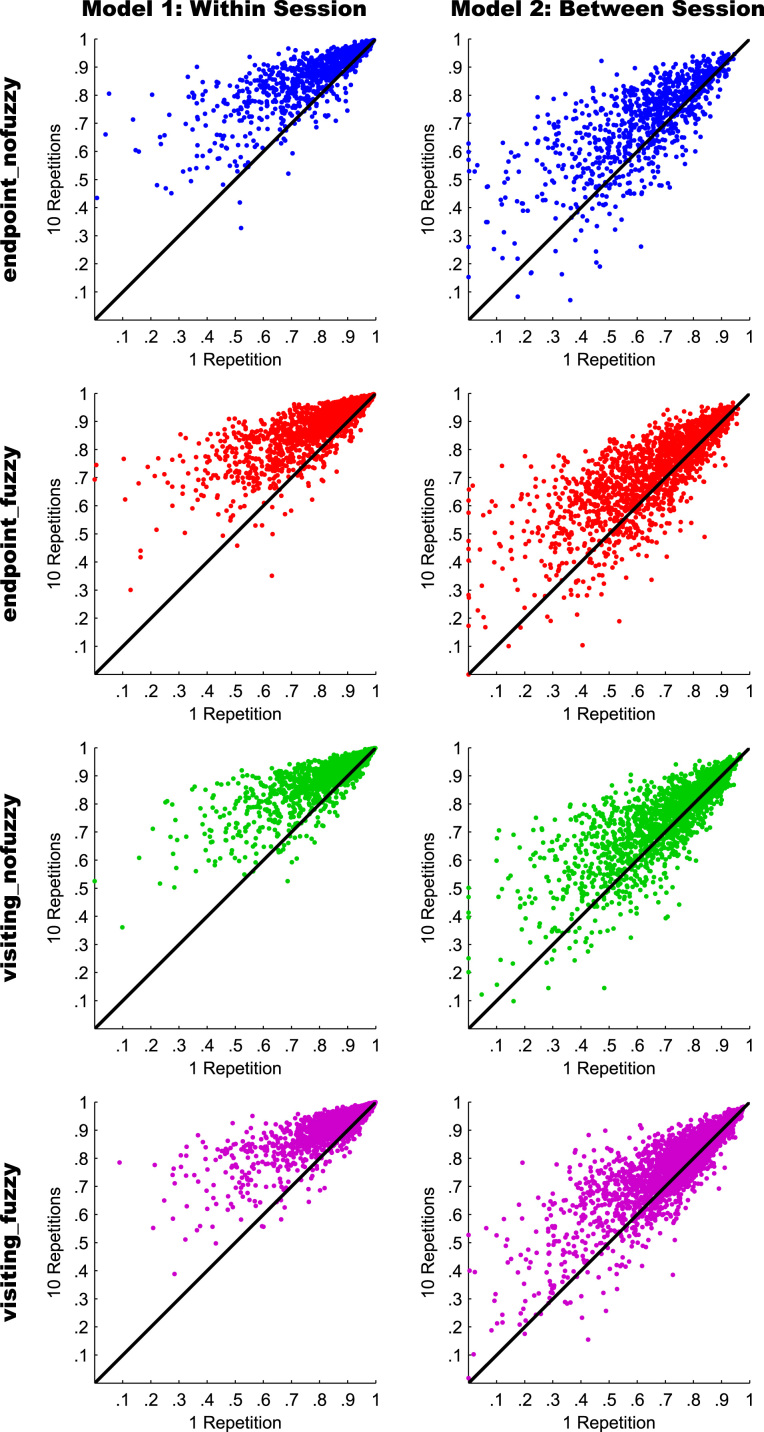
Scatterplots of connection-specific ICC(2,1) values for Models 1 (within session reproducibility; left column) and 2 (between-session reproducibility; right column). In each column and for each streamline selection variant, the ICC(2,1) value for a given connection derived from 1 reconstruction repetition (*x*-axis) is plotted against the ICC(2,1) value for the same connection derived from 10 reconstruction repetitions (*y*-axis). Thus, connections with a higher ICC(2,1) value for 10 repetitions are above the diagonal, and connections with a higher ICC(2,1) value for 1 repetition are below the diagonal. For visualization purposes, negative ICC(2,1) values were set to zero (0.08% of values).

**Fig. 5:**
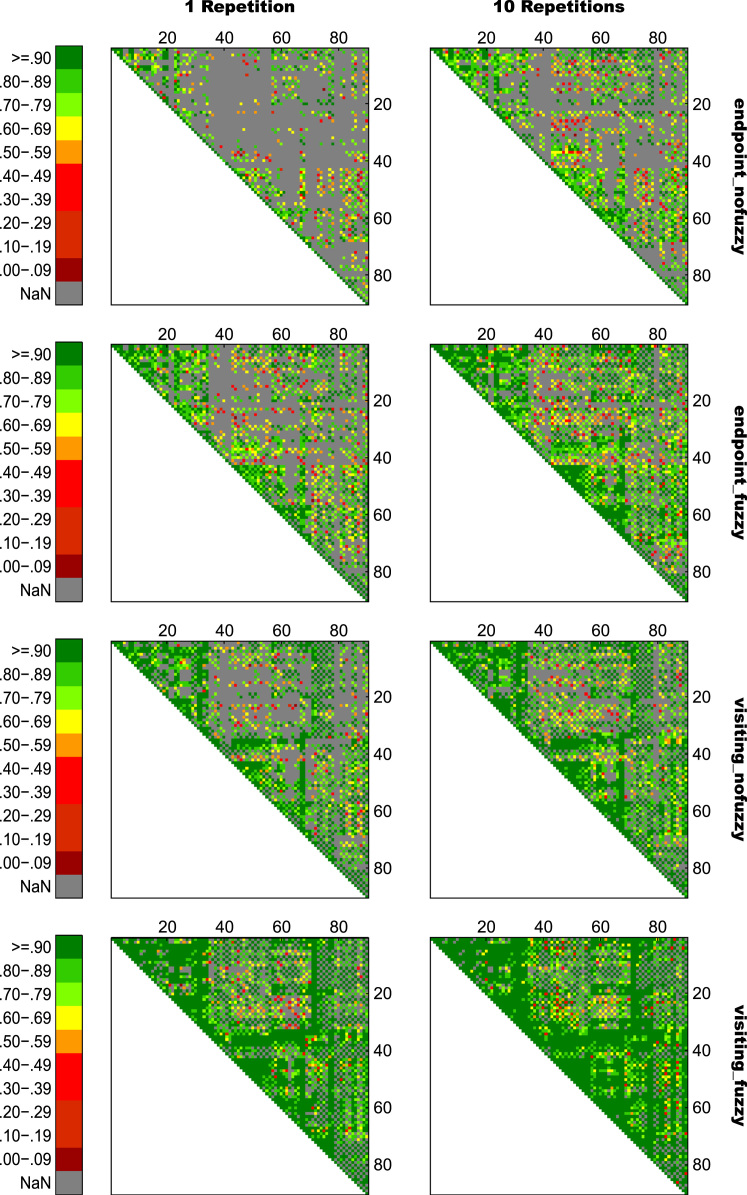
Seed-specific ICC(2,1) values for all connections of Model 1, estimating the within-session reproducibility of all eight variants of *reconstruction repetitions*×*streamline selection variant*. Numbers on the *x*- and *y*-axis refer to AAL seed numbers. For visualization purposes, negative ICC(2,1) values were set to zero (0.02% of values).

**Fig. 6:**
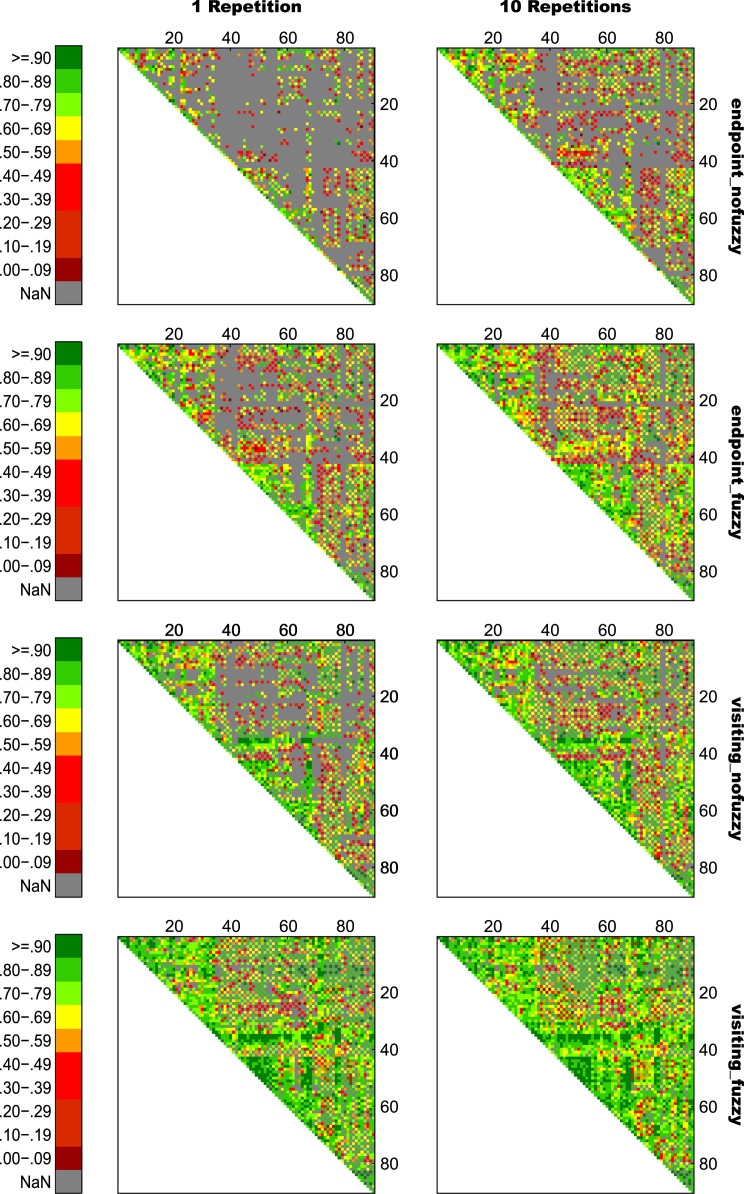
Seed-specific ICC(2,1) values for all connections of Model 2, estimating the between-session reproducibility of all eight variants of *reconstruction repetitions*×*streamline selection variant*. Numbers on the *x*- and *y*-axis refer to AAL seed numbers. For visualization purposes, negative ICC(2,1) values were set to zero (0.28% of values).

**Fig. 7:**
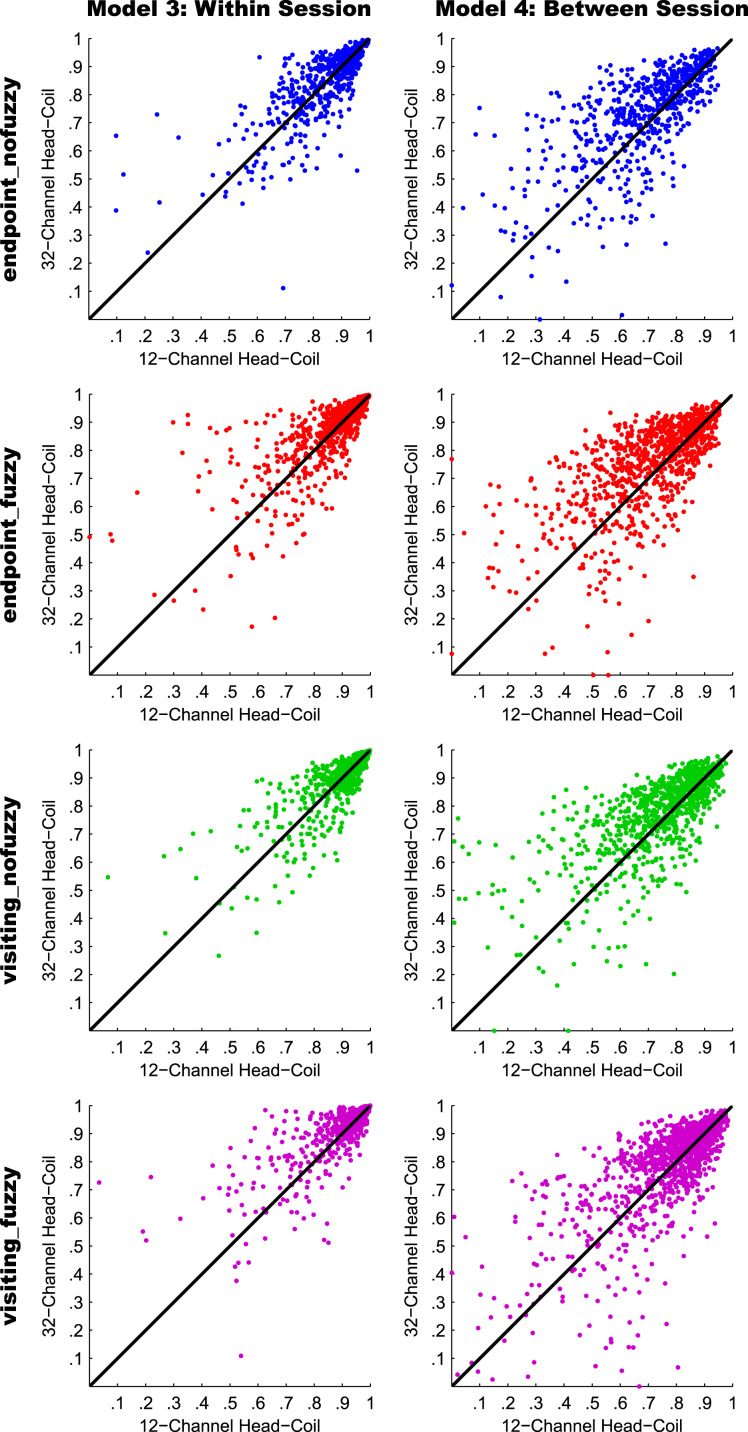
Scatterplots of connection-specific ICC(2,1) values for Models 3 (within-session reproducibility; left column) and 4 (between-session reproducibility; right column). In each column and for each streamline selection variant, the ICC(2,1) value for a given connection acquired with the 12-channel head-coil (*x*-axis) is plotted against the ICC(2,1) value for the same connection acquired with the 32-channel head-coil (*y*-axis). Thus, connections with a higher ICC(2,1) value for 32ch data are above the diagonal, and connections with a higher ICC(2,1) value for 12ch data are below the diagonal. For visualization purposes, negative ICC(2,1) values were set to zero (0.07% of values).

**Fig. 8:**
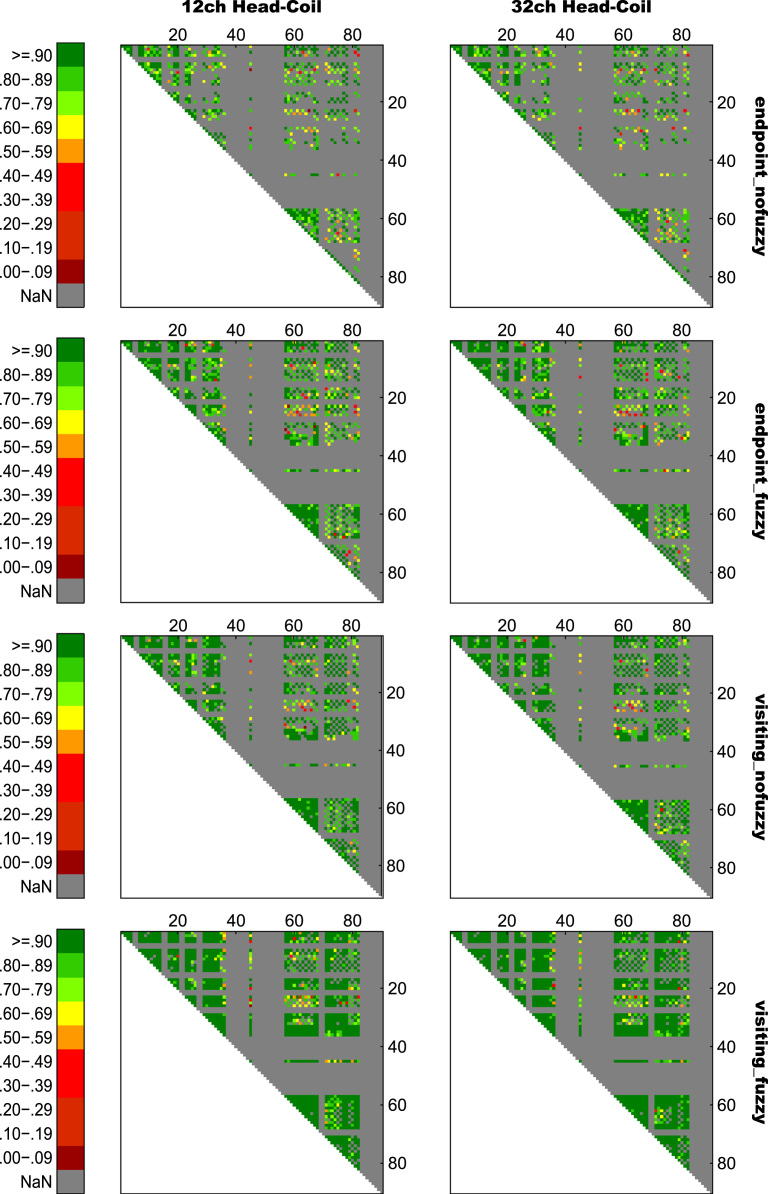
Seed-specific ICC(2,1) values for all connections of Model 3, estimating the within-session reproducibility of all eight variants of type of head-coil×streamline selection variant. Numbers on the *x*- and *y*-axis refer to AAL seed numbers. For visualization purposes, negative ICC(2,1) values were set to zero (0.01% of values).

**Fig. 9:**
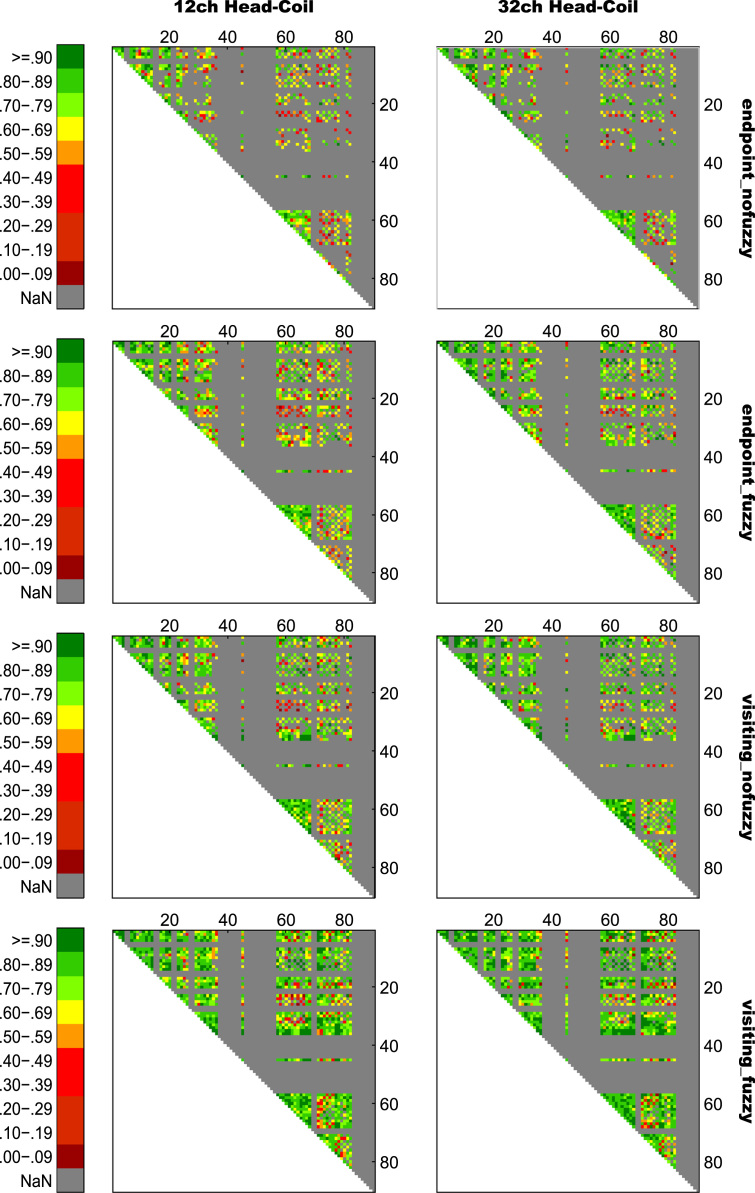
Seed-specific ICC(2,1) values for all connections of Model 4, estimating the between-session reproducibility of all eight variants of *type of head-coil*×*streamline selection variant*. Numbers on the *x*- and *y*-axis refer to AAL seed numbers. For visualization purposes, negative ICC(2,1) values were set to zero (0.13% of values).

For an overview on reproducibility data in terms of the intra-class correlation coefficient (ICC) for absolute agreement, the median ICC(2,1) values are given in Table 1. Table 2 shows the percentage of ICC(2,1) values <.60, corresponding to low reproducibility; between .60 and .69, corresponding to marginal reproducibility; and ≥.70, corresponding to adequate reproducibility or higher. Table 3 reports the reproducibility statistics for the four factorial models. These reproducibility statistics are further illustrated in terms of relative treatment effects (RTE) in Fig. 2. The entire raw ICC(2,1) values for all four factorial models are depicted in Fig. 3. In relation to factorial models 1 and 2, scatterplots in Fig. 4 illustrate the direct comparison of reproducibility for a given connection between tracking with 1 reconstruction repetition versus 10 repetitions, separately illustrated for the four selection variants. That is, for each streamline selection variant, the connections found in both the 1 repetition and 10 repetition data set are directly compared with each other. Information on seed-specific ICC(2,1) values is further presented in Fig. 5, Fig. 6 which depict the ICC values in a 90×45 connectivity matrix. In relation to factorial models 3 and 4, scatterplots in Fig. 7 depict connection-specific direct comparisons of reproducibility for 12ch- versus 32ch-coil data, separately illustrated for the four selection variants. Seed-specific ICC(2,1) values for the different combinations of type of head-coil and streamline selection variant are illustrated in 90×45 connectivity matrices in Fig. 8, Fig. 9.

**Table 1:** Median ICC(2,1) values for the four statistical models.

	Reconstruction Repetitions × Streamline Selection Variant				Type of Head-Coil × Streamline Selection Variant			
	Model 1:Within-Session Reproducibility		Model 2: Between-Session Reproducibility		Model 3: Within-Session Reproducibility		Model 4: Between-Session Reproducibility	
Streamline Selection Variant	1 Rep	10 Rep	1 Rep	10 Rep	12ch	32ch	12ch	32ch
	*Md*	*Md*	*Md*	*Md*	*Md*	*Md*	*Md*	*Md*
End_nofuz	.808	.828	.661	.663	.893	.899	.727	.772
End_fuz	.851	.883	.689	.700	.925	.929	.749	.794
Vis_nofuz	.911	.923	.741	.748	.952	.956	.784	.814
Vis_fuz	.954	.964	.793	.803	.981	.980	.823	.841

Numbers in cells refer to the median (*Md*) of intra-class correlation coefficient (ICC) type ICC(2,1) as per naming convention by Shrout and Fleiss [8] estimating the absolute agreement between measurements. 1 Rep, 1 reconstruction repetition; 10 Rep, 10 reconstruction repetitions; 12ch, 12-channel head-coil; 32ch, 32-channel head-coil; end_nofuz, endpoint_nofuzzy; end_fuz, endpoint_fuzzy; vis_nofuz, visiting_nofuzzy; and vis_fuz, visiting_fuzzy streamline selection.

**Table 2:** Percentage distribution of ICC(2,1) values for the four statistical models.

	Model 1: Within-Session Reproducibility						Model 2: Between-Session Reproducibility					
	1 Rep			10 Rep			1 Rep			10 Rep		
	ICC(2,1)			ICC(2,1)			ICC(2,1)			ICC(2,1)		
	<.60	.60 –.69	≥.70	<.60	.60–.69	≥.70	<.60	.60–.69	≥.70	<.60	.60–.69	≥.70
	%	%	%	%	%	%	%	%	%	%	%	%
End_nofuz	31.67	13.73	54.60	24.22	11.07	64.71	52.30	19.08	28.62	47.93	17.45	34.62
End_fuz	21.02	12.46	66.52	16.60	8.93	74.47	44.91	15.94	39.15	38.87	18.21	42.92
Vis_nofuz	14.11	8.56	77.34	11.76	7.64	80.60	34.42	15.92	49.67	30.57	16.80	52.63
Vis_fuz	9.31	4.65	86.04	8.60	4.80	86.60	21.33	12.56	66.11	20.63	11.63	67.74

	Model 3: Within-Session Reproducibility						Model 4: Between-Session Reproducibility					
	12ch			32ch			12ch			32ch		
	ICC(2,1)			ICC(2,1)			ICC(2,1)			ICC(2,1)		
	<.60	.60 –.69	≥.70	<.60	.60 –.69	≥.70	<.60	.60 –.69	≥.70	<.60	.60 –.69	≥.70
	%	%	%	%	%	%	%	%	%	%	%	%
End_nofuz	15.03	7.80	77.18	12.77	7.46	79.77	36.39	17.91	45.69	29.37	14.63	56.01
End_fuz	11.90	6.89	81.21	10.99	5.33	83.68	33.63	17.06	49.31	25.18	12.59	62.23
Vis_nofuz	8.26	5.34	86.40	7.94	5.26	86.80	26.81	14.81	58.37	21.18	12.72	66.10
Vis_fuz	6.47	3.59	89.94	4.42	3.01	92.57	18.22	10.87	70.91	16.30	9.59	74.10

For each combination of reconstruction repetition or type of head-coil with streamline selection variant, numbers in cells refer to the percentage of intra-class correlation coefficient (ICC) type ICC(2,1) values falling within each of the following three categories: ICC(2,1) <.60, corresponding to low reproducibility; ICC(2,1) between .60 and .69, corresponding to marginal reproducibility; ICC(2,1) ≥.70, corresponding to adequate reproducibility or higher. 1 Rep, 1 reconstruction repetition; 10 Rep, 10 reconstruction repetitions; 12ch, 12-channel head-coil; 32ch, 32-channel head-coil; end_nofuz, endpoint_nofuzzy; end_fuz, endpoint_fuzzy; vis_nofuz, visiting_nofuzzy; and vis_fuz, visiting_fuzzy streamline selection.

**Table 3:** Reproducibility statistics for the four factorial models.

	*Reconstruction Repetitions×Streamline Selection Variant*							
	Model 1: Within-Session Reproducibility				Model 2: Between-Session Reproducibility			
Factor	*F*	*df*	*p*	*η^2^*	*F*	*df*	*p*	*η^2^*
Repetitions	2268.52	1, 821	<.0001	.0863	442.35	1, 828	<.0001	.0445
Selection Variant	2822.28	2.30, 1884.41	<.0001	.6548	901.92	2.46, 2039.63	<.0001	.4157
Repetitions*Selection Variant	49.49	2.86, 2346.96	<.0001	.0021	50.96	2.67, 2218.82	<.0001	.0043

	*Type of Head-Coil* × *Streamline Selection Variant*							
	Model 3: Within-Session Reproducibility				Model 4: Between-Session Reproducibility			
Factor	*F*	*df*	*p*	*η^2^*	*F*	*df*	*p*	*η^2^*
Coil	5.88	1, 573	.0153	.0004	110.67	1, 577	< .0001	.0357
Selection Variant	1885.70	2.29, 1310.40	<.0001	.7038	464.87	2.46, 1418.41	< .0001	.2931
Coil* Selection Variant	2.24	2.84, 1625.78	.084	.0002	14.34	2.81, 1621.08	< .0001	.0029

Table gives the ANOVA-type statistics of the nparLD factorial models (see Section 2 and [6] for more details). Repetitions, reconstruction repetitions [1 vs 10 repetitions]; Selection Variant, streamline selection variant [endpoint_nofuzzy, endpoint_fuzzy, visiting_nofuzzy, visiting_fuzzy]; Coil, type of head-coil [12ch vs 32ch]; df, numerator and denominator degrees of freedom (separated by comma); *η*^2^, eta squared, denoting the share of total variance explained by each factor. As per convention by Cohen [14], an effect is considered small if *η*^2^≥.01, medium if *η*^2^≥.06, and large if *η*^2^≥.14.

**Fig. 10:**
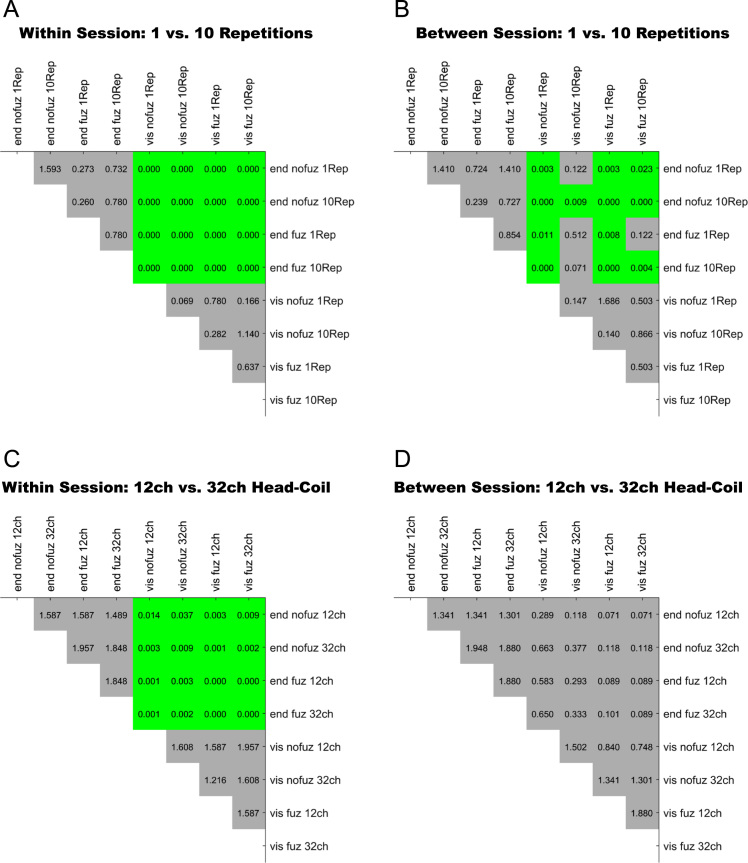
Pairwise tests of differences in the Spearman׳s rho correlation coefficients of ICC(2,1) values and mean seed size between all eight factor combinations for (A) Model 1, (B) Model 2, (C) Model 3, and (D) Model 4. Numbers in cells refer to *p*-values of the z-test of difference between the two Fisher׳s r-to-Z transformed correlation coefficients, adjusted for the false discovery rate (FDR) and then tested against *α*=.05. Please note that FDR-adjusted *p*-values can be larger than 1. Green shading denotes *p*<.05, i.e. significant differences between the two correlations. Gray shading denotes *p*>.05, i.e. non-significant differences between the two correlations. For all significant comparisons between a visiting and an endpoint variant, the correlation with mean seed size was significantly larger for the endpoint than the visiting variant (see Table 4). Abbreviations: End, endpoint streamline selection; vis, visiting streamline selection; nofuz, no fuzzy streamline selection; fuz, fuzzy streamline selection; 1 Rep, 1 reconstruction repetition; 10 Rep, 10 reconstruction repetitions; 12ch, 12-channel head-coil; 32ch, 32-channel head-coil.

**Fig. 11:**
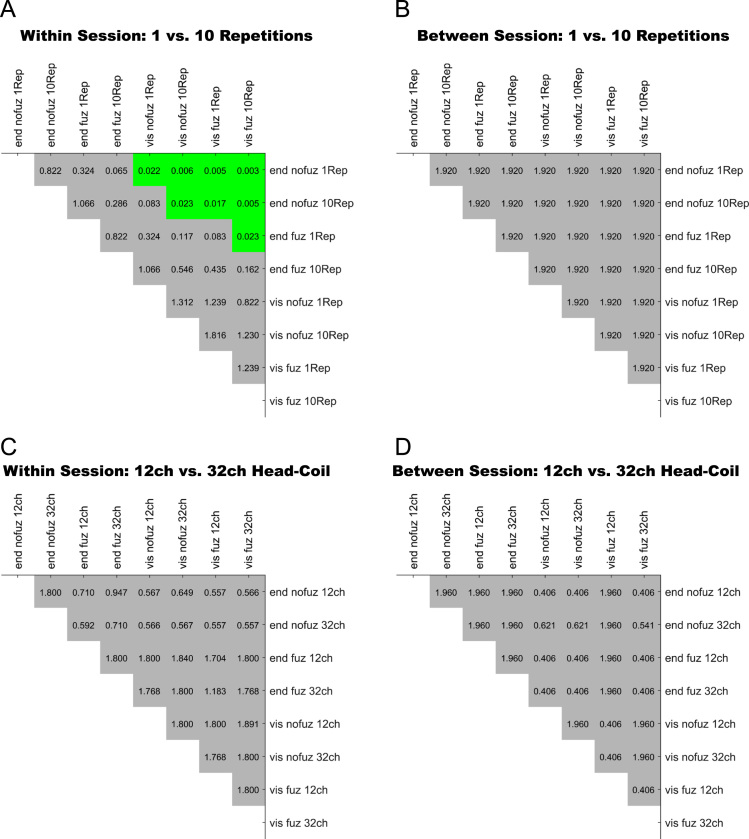
Pairwise tests of differences in the Spearman׳s rho correlation coefficients of ICC(2,1) values and mean streamline counts between all eight factor combinations for (A) Model 1, (B) Model 2, (C) Model 3, and (D) Model 4. Numbers in cells refer to *p*-values of the z-test of difference between the two Fisher׳s r-to-Z transformed correlation coefficients, adjusted for the false discovery rate (FDR) and then tested against *α*=.05. Please note that FDR-adjusted *p*-values can be larger than 1. Green shading denotes *p*<.05, i.e. significant differences between the two correlations. Gray shading denotes *p*>.05, i.e. non-significant differences between the two correlations. For all significant comparisons between a visiting and an endpoint variant, the correlation with mean streamline counts was significantly larger for the visiting than the endpoint variant (see Table 4). Abbreviations: End, endpoint streamline selection; vis, visiting streamline selection; nofuz, no fuzzy streamline selection; fuz, fuzzy streamline selection; 1 Rep, 1 reconstruction repetition; 10 Rep, 10 reconstruction repetitions; 12ch, 12-channel head-coil; 32ch, 32-channel head-coil.

Table 4 reports the correlation of ICC(2,1) values of all factor combinations of the four factorial models with mean seed size and mean streamline counts. Figs. 10 and 11 depict FDR-adjusted *p*-values of the tests for differences between the correlations of all factor combinations of each factorial model.

**Table 4:** Correlations of ICC(2,1) values with mean seed size and mean streamline counts.

	Reconstruction Repetitions × Streamline Selection Variant				Type of Head-Coil × Streamline Selection Variant			
	Model 1:Within-Session Reproducibility		Model 2: Between-Session Reproducibility		Model 3: Within-Session Reproducibility		Model 4: Between-Session Reproducibility	
Streamline Selection Variant	1 Rep	10 Rep	1 Rep	10 Rep	12ch	32ch	12ch	32ch
	*ρ*	*ρ*	*ρ*	*ρ*	*ρ*	*ρ*	*ρ*	*ρ*
*Correlation of ICC(2,1) with mean seed size*								
End_nofuz	.335	.326	.314	.329	.307	.333	.306	.275
End_fuz	.271	.298	.273	.299	.333	.340	.275	.270
Vis_nofuz	.075^a^	.146	.175	.234	.172	.171	.211	.189
Vis_fuz	.102	.130	.180	.213	.139	.160	.162	.157

*Correlation of ICC(2,1) with mean streamline counts*								
End_nofuz	.723	.767	.623	.614	.759	.748	.619	.601
End_fuz	.794	.826	.641	.629	.841	.827	.621	.609
Vis_nofuz	.851	.865	.615	.596	.856	.846	.526	.527
Vis_fuz	.868	.882	.626	.606	.876	.859	.618	.524

Numbers in cells refer to Spearman׳s rho (*ρ*) coefficient of the correlation between intra-class correlation coefficient type ICC(2,1) and the mean seed size or mean streamline count of each connection across all connections of each of the four statistical models. The number of voxels per AAL seed ranged from 220 (seed 41, left amygdala) to 5104 (seed 8, right middle frontal gyrus). Effect sizes for correlations were considered small if *ρ*≥.10, medium if *ρ*≥.30, and large if *ρ*≥.50 [2]. Except if otherwise noted, correlations were significant at an FDR-corrected threshold of *p*<.001. 1 Rep, 1 reconstruction repetition; 10 Rep, 10 reconstruction repetitions; 12ch, 12-channel head-coil; 32ch, 32-channel head-coil; end_nofuz, endpoint_nofuzzy; end_fuz, endpoint_fuzzy; vis_nofuz, visiting_nofuzzy; and vis_fuz, visiting_fuzzy streamline selection.

a FDR-corrected *p*=.004.

## 2 Experimental design, materials and methods

### 2.1 Participants

All participants who provided data were students recruited from the University of Freiburg who participated voluntarily. To be eligible for participation, participants had to have German as native language, normal or corrected-to-normal vision, unimpaired color vision and be neurologically and psychiatrically healthy. Based on these inclusion criteria, data from a total of 30 participants, who gave written informed consent to participation and were compensated with 60€, were collected. Two participants had to be excluded because of a high depressivity score and an incidental MRI finding, leaving data from *N*=28 participants (*N*=13 males) with a mean (±SD) age of 22.53 (±1.77) years and a mean 15.82 (±1.54) years of education for analysis.

Data collection was approved by the local ethics committee and conducted in accordance with the Declaration of Helsinki. Please note that this sample is identical to the one in the related research article (Schumacher et al., Probing the reproducibility of quantitative estimates of structural connectivity derived from global tractography). Further details on the sample can be found in the related research article.

### 2.2 Magnetic resonance imaging

In two separate testing sessions, MR imaging was performed on the same 3T TimTrio MR scanner (Siemens GmbH, Erlangen, Germany) acquiring the same set of MRI data. Using a 12-channel head-coil, the following imaging sequences were acquired: a three-dimensional T1-weighted magnetization-prepared rapid gradient-echo (MPRAGE) sequence (repetition time [TR], 2200 ms; echo time [TE], 2.15 ms; flip angle, 12°; 160 sagittal slices; matrix size, 256×256; field of view, 256 mm; voxel size, 1×1×1 mm^3^) and a diffusion-sensitive single-shot spin-echo echo-planar imaging (EPI) sequence with cerebrospinal fluid (CSF) suppression applying a HARDI (high angular resolution diffusion imaging) acquisition scheme with 61 diffusion encoding gradient directions (b-factor, 1000 s/mm^2^); 69 axial slices; TR, 10 000 ms; TE, 94 ms; flip angle, 90°; matrix size, 104×104; field of view, 208 mm; voxel size, 2×2×2 mm^3^ and nine scans without diffusion weighting (b-factor, 0 s/mm^2^), equally distributed across the acquisition series. A 32-channel head-coil was used to acquire a second diffusion-sensitive EPI sequence with 61 diffusion encoding gradient directions (b-factor, 1000 s/mm^2^); 43 axial slices; TR, 6500 ms; TE, 94 ms; matrix size, 104×104; field of view, 208 mm; voxel size, 2×2×2 mm^3^ in addition to nine images with a b-factor of 0 s/mm^2^. An in-house motion and artifact correction algorithm was applied to all imaging sequences during image reconstruction [2]. Individual T1-weighted MPRAGE images were segmented and normalized with the Voxel Based Morphometry Toolbox (VBM8; version 435, http://dbm.neuro.uni-jena.de) incorporated in SPM8 (release r5236; http://www.fil.ion.ucl.ac.uk/spm/software/spm8), running on MATLAB 7.14.0.739 (R2012a; The Mathworks, Inc., Natick, MA, USA) using the default options and the DARTEL (diffeomorphic anatomical registration through exponentiated lie algebra) approach [3] for spatial normalization into the Montreal Neurological Institute (MNI) stereotaxic space with non-linear warping only, applying the DARTEL template derived from *N*=550 healthy subjects of the IXI database (http://brain-development.org/ixi-dataset). Preprocessing of diffusion-weighted images comprised calculation of the diffusion tensors [4] from the motion- and distortion-corrected images using the in-house *DTI & Fibertools* toolbox (version 20141029; https://www.uniklinik-freiburg.de/mr-en/research-groups/diffperf/fibertools.html) implemented in SPM8.

### 2.3 Global fiber tractography

Tractography based on HARDI images was performed using the global tractography approach or Gibbs tracking implemented in DTI & Fibertools [1]. With this approach, the entire connectome of estimated fibers is reconstructed in a single optimization step by modeling segments of the to-be-reconstructed fibers as small cylinders freely moving in the tissue due to Brownian motion. During an iterative simulated annealing procedure, the assumed temperature is slowly reduced so that the cylinders align to form longer chains and then fiber tracts [1]. To designate the white-matter voxels considered for fiber reconstruction, each individual׳s whole-brain white matter image from the first testing session (segmented, written in native space, co-registered and resliced with reference to the b0-image, binarized at a threshold of >.50) was used. Fiber reconstruction was carried out using the ‘dense’ parameter set (segment weight of 0.05, corresponding to the percentage of brain-averaged anisotropic signal at which reconstruction of fibers was thresholded; a start and stop temperature of 0.1 and 0.001, respectively; 5×10^8^ iterations; and a minimum fiber length of 10 cylinder segments; cf. [1]).

In addition to this standard procedure, we additionally applied a technique whereby a repetitive reconstruction of fibers is introduced. After the initial global tracking is completed, the system is set to a given temperature at which a repeated number of ‘samples’ are collected. In detail, the end state of the first estimation process (i.e. repetition) represents the start state for the next estimation process, with the iterative annealing procedure being started anew from this start state. The end state of that annealing procedure is then again used as start state for the next repetition and so forth. The streamlines reconstructed during each repetition are then sum-aggregated into a single fiber tracking output file. For this repetitive reconstruction, the number of repetitions, a temperature at which samples are collected, and the number of iterations for the annealing procedure applied during every repetition have to be given. Here we used 1×108 iterations at a temperature of 0.1 (effectively resulting in complete repetition of the whole fiber reconstruction procedure).

### 2.4 Streamline selection

To select streamlines, binary seed images were re-normalized from MNI space into an individual׳s native space to select the subject-specific streamlines. This selection procedure demands that two parameters be defined: First, either streamlines ending in the seed or streamlines passing through the seed can be selected (*endpoint vs. visiting streamlines*). Second, seed images can either be used in their binary version, so that only seed mask voxels with a value of 1 are considered for streamline selection, or a Gaussian kernel is applied to the seed image voxels, resulting in ‘fuzzy’ borders of the image mask, so that streamline selection is then based on voxels that have at least a given minimum probability of being inside the seed mask (*fuzzy vs. no fuzzy selection*). Here we used a Gaussian kernel with *σ*=1 mm (corresponding FWHM ≈ 2.35 mm) and a minimum probability of 0.1 for fuzzy selection. These two selection parameters result in four possible variants for the selection of streamlines: endpoint_nofuzzy, endpoint_fuzzy, visiting_nofuzzy, and visiting_fuzzy.

The streamlines for the single seeds (i.e. all streamlines that end in/pass through a seed) and for all pairwise connections between the 90 seeds were selected using the four streamline selection variants. As streamline counts are estimated without directional information, there were 4095 possible streamline count values in total (89×45 bivariate connections plus the 90 single-seed streamline counts). The streamline counts for a given connection were divided by the subject-specific total number of streamlines (computed for each unique combination of testing session, type of head-coil, tracking repetition, and tracking run) to correct for differences in head size, which influence the total number of streamlines reconstructed [5]. This adjusted number of streamlines was multiplied by the sample mean of total streamline counts to yield a value in the same magnitude order as the uncorrected number of streamlines. For a connection to be included in the statistical analyses, an adjusted streamline count greater than 0 had to be present in at least 95% of subjects in both measurements on which reproducibility was computed. For the connections passing this threshold, a streamline count of zero was given if no streamlines had been reconstructed for a subject. Data acquisition with the 32ch head-coil resulted in volumes with 43 slices only partly covering the temporal, inferior frontal, and inferior occipital lobes. A seed was considered sufficiently covered if at most 5% of its voxels were outside of the 32ch-coverage mask (mask image of the individual head-coil placement and coverage). Across subjects and sessions (*N*=56), a seed was considered for analysis if sufficiently covered in at least 95% of subjects. This procedure resulted in 37 seeds being excluded from analysis for the 32ch-coil data: 5, 6, 15, 16, 21, 22, 27, 28, 37–44, 46–56, 69, 70, 83–90 (AAL numbering convention).

For an indication of how many of all reconstructed streamlines actually entered subsequent statistical analyses, Table 5 reports the percentage of total streamline counts to which streamline counts of selected connections corresponded (i.e. counting the streamlines that ended in both gray matter seeds forming a given connection relative to all reconstructed streamlines). This was carried out for within-session and between-session reproducibility analysis of 12ch head-coil data. In detail, the adjusted number of streamlines for all bivariate connections present in at least 95% of subjects across both tracking runs (within-session reproducibility) or both testing sessions (between-session reproducibility) were sum-aggregated and divided by the total number of streamlines per subject. This percentage value was mean-aggregated across subjects. To avoid redundant counting of streamlines due to visiting or fuzzy selection, only data of the endpoint_nofuzzy streamline selection were used. As the 32ch head-coil did not provide whole-brain coverage and thus selection of streamlines was not based on all seeds of the AAL atlas (see above), 32ch data were not used for this calculation. On average, roughly 50% of all reconstructed streamlines entered statistical analyses for the endpoint_nofuzzy streamline selection variant (Table 5).

**Table 5:** Percentage of total number of streamlines selected for statistical analysis using endpoint_nofuzzy streamline selection for data acquired with the 12-channel head-coil.

	Within-Session Reproducibility				Between-Session Reproducibility			
	Session 1				Session 1		Session 2	
	Run 1		Run 2		Run 1		Run 1	
	*M (SD)*	*Min; Max*	*M (SD)*	*Min; Max*	*M (SD)*	*Min; Max*	*M (SD)*	*Min; Max*
1 Rep	49.68 (7.41)	36.42; 66.03	49.65 (7.44)	36.44; 65.91	49.73 (7.40)	36.52; 66.07	49.60 (7.09)	35.71; 65.40
10 Rep	54.96 (8.06)	40.72; 72.77	54.94 (8.01)	40.78; 72.36	54.99 (8.06)	40.79; 72.82	54.86 (7.69)	40.20; 71.56

Within-session reproducibility refers to data acquired with the 12ch head-coil using the criterion that for a given connection a streamline count larger than 0 had to be present in at least 95% of subjects on both tracking runs of the first testing session. Between-session reproducibility refers to data acquired with the 12ch head-coil using the criterion that for a given connection a streamline count larger than 0 had to be present in at least 95% of subjects on the first tracking run of both testing sessions. 1 Rep, 1 reconstruction repetition; 10 Rep, 10 reconstruction repetitions.

### 2.5 Experimental design and statistical analysis

A schematic of the experimental design is depicted in Fig. 1. Independent diffusion-weighted image data were not only acquired on two *testing sessions*, but were also acquired with two different *types of head-coil* (12 channels [12ch] vs 32 channels [32ch]). In addition, global tractography was performed twice on the data from each session, thus resulting in two independent *runs of tracking* per testing session and type of head-coil, and was furthermore separately performed with two variants of *reconstruction repetitions* (1 repetition vs. 10 repetitions). In addition, the selection of streamlines was carried out using the four *streamline selection variants*. Thus, for a given AAL-based connection in an individual, there were 64 independent streamline count estimations: 2 *sessions*×2 *types of head-coil*×2 *tracking runs*×2 *variants of reconstruction repetitions*×4 *streamline selection variants* (Fig. 1). For connections not included in the 32ch-coil data due to insufficient seed coverage, there were 32 independent streamline count estimations.

Statistical analyses assessed the impact of the following three factors on the reproducibility of streamline counts: (i) *streamline selection variant* (endpoint_nofuzzy, endpoint_fuzzy, visiting_nofuzzy, visiting_fuzzy), (ii) *number of reconstruction repetitions* (1 vs. 10 repetitions), and (iii) *type of head-coil* (12ch vs 32ch). As data for some factor combinations were non-normally distributed, non-parametric analyses were run with the R statistics package *nparLD* [6] on the ranks of values (instead of the raw values), thus constituting a non-parametric equivalent to a repeated-measures analysis of variance (ANOVA). However, nparLD is restricted to test the effects of a maximum of two within-subject and two between-subjects factors at once so that the three factors of interest here could not be evaluated in one overall model but four separate models.

In consequence, only the 12ch-coil data were used to assess the effect of reconstruction repetitions and only the data from 10 reconstruction repetitions were used to compare the 12ch- and 32ch-coil data. This procedure yielded two analytical designs with two factors each: *reconstruction repetitions*×*streamline selection variant* and *type of head-coil*×*streamline selection variant*. For both analytical designs, reproducibility was assessed for (a) *within-session data* by comparing the streamline counts from the two tracking runs of the first testing session and for (b) *between-session data* by comparing streamline counts between the two testing sessions (using the data from the first tracking run of each testing session). Thus, four reproducibility analyses were performed (Fig. 1): Model 1, within-session reproducibility of *reconstruction repetitions*×*streamline selection variant*; Model 2, between-session reproducibility of *reconstruction repetitions*×*streamline selection variant*; Model 3, within-session reproducibility of *type of head-coil*×*streamline selection variant*; Model 4, between-session reproducibility of *type of head-coil* ×*streamline selection variant*.

For each of these four analyses, reproducibility was assessed connection-wise using intra-class correlation coefficients (ICC) based on two-way random effects models [7], [8], with participants as “targets” and the two measurements (i.e., the two tracking runs or the two testing sessions) as “raters”. That is, for each connection, the between-subjects and within-subject difference in streamline counts was assessed to probe the amount of total variance that can be attributed to true interindividual differences [8], [9]. Here we assessed the *absolute agreement* [10], that is, the absolute difference in individual streamline count estimates between measurements, corresponding to ICC(2,1) according to Shrout and Fleiss (1979) (or ICC(A,1) according to McGraw and Wong, 1996). Reproducibility was considered low if ICC(2,1) ≤ .59, marginal for .60–.69, adequate for .70–.79, high for .80–.89, and very high if ≥ .90 [11].

Factorial models were then computed on ICC(2,1) values to evaluate the four models described above using the nparLD package (version 2.1; [6]) for *R* statistics (version 3.3.1;[12]). For Model 1, there were *N*=822 bivariate connections that were found in all of the eight data sub-sets. Thus, their respective ICC(2,1) values entered the model as “subjects”. For Model 2, *N*=829 bivariate connections were successfully reconstructed in all eight factor combinations and thus their ICC(2,1) values entered the nparLD analysis. For Model 3, the ICC(2,1) values of *N*=574 bivariate connections entered the within-session model with type of head-coil and streamline selection variant as within-subject factors. In Model 4, the between-session analysis on ranks for ICC(2,1) values with head-coil and selection variant as within-subject factors was based on *N*=578 connections.

Furthermore, the dependency of ICC(2,1) values on the size of the seeds used for streamline selection and on the streamline counts themselves was probed. To this end, for each connection, the mean number of voxels of the two seed masks and the mean streamline count value (aggregated across the sample and both tracking runs or testing sessions, i.e. *N*=56) were computed and correlated with the ICC(2,1) value of that connection using Spearman׳s rho rank correlation, with ICC(2,1) values being Fisher r-to-Z transformed beforehand. Correlations were computed for each factorial combination in the four statistical models separately. Subsequently, it was tested whether there were significant differences between the eight correlation coefficients per statistical model. To this end, Spearman correlation coefficients were Fisher r-to-Z transformed; all possible differences between the correlations were computed and compared against the standard normal z-distribution. To correct for multiple testing, *p*-values were adjusted for the false discovery rate (FDR) according to Benjamini and Yekutieli [13] within each of the statistical models and then tested against a significance threshold of *α*=.001 for the correlation coefficients themselves (*N*=8 tests per model) and a threshold of *α*=.05 for the pairwise differences in correlation coefficients (*N*=28 tests per model). FDR correction was performed in Matlab using the fdr_bh script by David Groppe (available from www.mathworks.com/matlabcentral/fileexchange/27418-fdr-bh/content/fdr_bh.m; accessed on 20th September 2016).

## References

[1] Reisert M., Mader I., Anastasopoulos C., Weigel M., Schnell S., Kiselev V. (2011). Global fiber reconstruction becomes practical. Neuroimage.

[2] Zaitsev M., Hennig J., Speck O. (2004). Point spread function mapping with parallel imaging techniques and high acceleration factors: fast, robust, and flexible method for echo-planar imaging distortion correction. Magn. Reson. Med..

[3] Ashburner J. (2007). A fast diffeomorphic image registration algorithm. Neuroimage.

[4] Basser P.J., Mattiello J., Lebihan D. (1994). Estimation of the effective self-diffusion tensor from the NMR spin echo. J. Magn. Reson. Ser. B.

[5] Kaller C.P., Reisert M., Katzev M., Umarova R., Mader I., Hennig J., Weiller C., Köstering L. (2015). Predicting planning performance from structural connectivity between left and right mid-dorsolateral prefrontal cortex: moderating effects of age during postadolescence and midadulthood. Cereb. Cortex.

[6] Noguchi K., Gel Y.R., Brunner E., Konietschke F. (2012). nparLD: an R software package for the nonparametric analysis of longitudinal data in factorial experiments. J. Stat. Softw..

[7] Shrout P.E., Fleiss J.L. (1979). Intraclass correlations: uses in assessing rater reliability. Psychol. Bull..

[8] Weir J.P. (2005). Quantifying test-retest reliability using the intraclass correlation coefficient and the SEM. J. Strength Cond. Res..

[9] Lachin J.M. (2004). The role of measurement reliability in clinical trials. Clin. Trials.

[10] McGraw K.O., Wong S.P. (1996). Forming inferences about some intraclass correlations coefficients. Psychol. Methods.

[11] Strauss E., Sherman E., Spreen O. (2006). A Compendium of Neuropsychological Tests: Administration, Norms, and Commentary.

[12] R Core Team, R: A Language and Environment for Statistical Computing, 2016. 〈http://www.r-project.org〉.

[13] Benjamini Y., Yekutieli D. (2001). The control of the false discovery rate in multiple testing under dependency. Ann. Stat..

[14] Cohen J. (1988). Statistical Power Analysis for the Behavioral Sciences.

