# Supplementary material for: Data on the test-retest reproducibility of streamline counts as a measure of structural connectivity
Source: Data Brief. 2018 Jun 5;19:1361–81. doi: 10.1016/j.dib.2018.05.145 (PMC6140827; doi:10.1016/j.dib.2018.05.145)
Supplement: Supplementary file 1 — Supplementary material [file mmc1.pdf]

Conflicts of Interests

The authors of this article (LVS, MR, KN, KE, HU, JH, CW, CPK) report no conflicts of interest.

The present research was supported by a grant of the BrainLinks-BrainTools Cluster of Excellence funded by the German Research Foundation (DFG; grant # EXC 1086) and by a grant of the Clinical Trials Programme of the Faculty of Medicine, University of Freiburg. LVS received PhD scholarship funds from the State Graduate Funding Program of Baden-Württemberg, Germany.

The funding sources had no involvement in the design and collection of data or the writing of the manuscript.

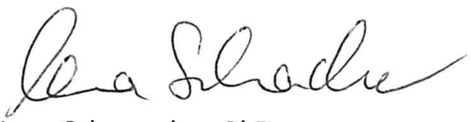

Lena Schumacher, PhD
on behalf of all authors

Freiburg 18/04/2018

lena.schumacher@mps.uni-freiburg.de
